# Closed-Loop Temperature Control Based on Fiber Bragg Grating Sensors for Laser Ablation of Hepatic Tissue

**DOI:** 10.3390/s20226496

**Published:** 2020-11-13

**Authors:** Sanzhar Korganbayev, Annalisa Orrico, Leonardo Bianchi, Martina De Landro, Alexey Wolf, Alexander Dostovalov, Paola Saccomandi

**Affiliations:** 1 Department of Mechanical Engineering, Politecnico di Milano Milan, 20133 Milano MI, Italy; annalisa.orrico@mail.polimi.it (A.O.); leonardo.bianchi@polimi.it (L.B.); martina.delandro@polimi.it (M.D.L.); paola.saccomandi@polimi.it (P.S.); 2 Laboratory of Fiber Optics, Institute of Automation and Electrometry SB RAS, Novosibirsk 630090, Russia; wolf@iae.nsk.su (A.W.); dostovalov@iae.nsk.su (A.D.)

**Keywords:** laser ablation, thermal ablation, temperature measurements, optical fiber, fiber Bragg grating sensors, feedback system, real-time monitoring, closed-loop temperature control

## Abstract

Laser ablation (LA) of cancer is a minimally invasive technique based on targeted heat release. Controlling tissue temperature during LA is crucial to achieve the desired therapeutic effect in the organs while preserving the healthy tissue around. Here, we report the design and implementation of a real-time monitoring system performing closed-loop temperature control, based on fiber Bragg grating (FBG) spatial measurements. Highly dense FBG arrays (1.19 mm length, 0.01 mm edge-to-edge distance) were inscribed in polyimide-coated fibers using the femtosecond point-by-point writing technology to obtain the spatial resolution needed for accurate reconstruction of high-gradient temperature profiles during LA. The zone control strategy was implemented such that the temperature in the laser-irradiated area was maintained at specific set values (43 and 55 °C), in correspondence to specific radii (2 and 6 mm) of the targeted zone. The developed control system was assessed in terms of measured temperature maps during an ex vivo liver LA. Results suggest that the temperature-feedback system provides several advantages, including controlling the margins of the ablated zone and keeping the maximum temperature below the critical values. Our strategy and resulting analysis go beyond the state-of-the-art LA regulation techniques, encouraging further investigation in the identification of the optimal control-loop.

## 1. Introduction

Many thermal ablation (TA) therapies are being proposed as alternatives to the traditional cancer treatment methods, e.g., resectional surgery, chemotherapy, and radiation therapy, for the treatment of non-surgical patients [1]. All TA techniques are based on localized temperature change that is created to induce the malignant cell necrosis in the ablated tumor at cytotoxic temperatures (50−60 °C) [2]. The main advantage of TA treatment over conventional treatment techniques is TA’s minimal invasiveness, as, for instance, small-size applicators can be used under percutaneous or endoscopic guidance to induce local temperature change [3,4,5]. Depending on the frequency of electromagnetic waves that induce tissue’s temperature change, TA techniques are divided into high-intensity focused ultrasound [6], microwave [7], radiofrequency [8], and laser ablation (LA) [9].

Among all these techniques, LA holds good promises for clinical application, taking advantage of the small and flexible fiber optic applicators guiding laser energy into deep-lying tumors [10], which makes the procedure also safe and compatible with magnetic resonance imaging (MRI) and computed tomography (CT) techniques. Besides, the laser can be used for the treatment of superficial tumors, also inside of hollow organs, such as the and gastrointestinal mucosa [11,12], and biliary tree [13], and in combination with selectively absorbing nanomaterials for photothermal therapy [14,15]. LA’s efficacy has been already investigated for the treatment of tumors in several organs, including bones [16], brain [17], thyroid [18], liver [19], and pancreas [20,21,22].

However, the abovementioned advantages are not preponderating two main limitations of LA treatment. The first one is an inefficient ablation selectivity when healthy surrounding tissues can also be damaged. The second limitation is the use of an open-loop approach, i.e., laser parameters (power, position, and treated shape) are usually set before the procedure without modulation during the ablation [3]. One of the possible solutions to both problems is the development of an ablation controlling technique based on intra-tissue tumor parameters, such as temperature, strain, tissue’s refractive index, and other biological markers. Due to the nature of TA treatments, the temperature during ablation is the most important parameter among them that significantly affects treatment efficacy [23]. 

The complex phenomenon of the laser–tissue interaction (absorption, reflection, and scattering) and a small diameter of the laser-guiding fiber leads to a high spatial thermal gradient [24]. As a result, the ablated area has several regions with different biological effects that depend on the duration of the ablation and the maximum temperature reached. In this context, the temperature range between 42 and 45 °C is considered to be optimal for hyperthermal treatment since it activates immune responses by promoting the migration and maturation of Langerhans cells [25]. At a temperature between 50 to 55 °C, coagulative necrosis starts in organs [3], and it has been observed that cellular death can occur instantaneously in cell culture [26]. A temperature of 60 °C is the threshold for rapid protein denaturation, which leads to a cytotoxic effect and coagulative necrosis. In order to evaluate the effect of the ablation duration and the maximum temperature reached, i.e., relative treatment effectiveness, different models are proposed. For instance, thermal dose can be expressed as equivalent minutes of exposure at 43 °C (Cumulative Equivalent Minutes at 43 °C, CEM43), which has been found to correlate with the severity of thermal damage for several tissue types [27]. Volumes of the regions with different thermal doses depend on complex combinations of different parameters, such as penetration depth of the laser light, shape of the applicator, absorption, mechanical and thermal properties of the tissue, etc. [23]. Hence, it is challenging to have effective pre-operative modeling of LA treatment and to obtain optimal settings of the laser parameters before the procedure. As a result, temperature monitoring in terms of both accuracy and spatial resolution plays a crucial role in effective LA treatment. 

Most of the works on real-time power regulation during LA use temperature as a primary parameter to control the ablation procedure. Ivarsson et al. developed temperature-controlled stepwise power regulation for LA of ex vivo bovine muscle experiments; the temperature was monitored using thermistors (10 mm spatial resolution) [28]. Möller and collaborators used ablation control based on 5-point thermistor probe temperature measurements for in vivo LA in rats and compared them with in vitro temperature control and light penetration experiments [29]. In [30], regulation is based on photo-optic probe temperature measurements during in vivo pig liver LA; the tissue necroses were evaluated with MRI after the treatment. Lin et al. employed thermocouple-based temperature measurements to regulate ablation [31,32]. 

State-of-the-art works, to the best of our knowledge, utilize conventional thermistor probes and thermocouple sensors that impair the accurate thermometry during LA. These sensors allow single-point measurements, thus restricting the number of sensors that can be simultaneously employed to measure and control the ongoing treatment to guarantee reduced invasiveness. Additionally, the laser light and heat absorption by metallic material of the probes can cause measurement artifact above 20 °C when the relative distance between applicator and sensor is small (a few millimeters) [33,34]. In this setup, the closer the distance between the metallic sensor and the applicator, the higher the overestimation due to the direct light absorption. This property forces to locate sensors far from the applicator tip (>1 cm), thus limiting the options for the control of the extension of the region under treatment. Both the mentioned problems lead to low spatial resolution thermometry, with potential sub-optimal and inefficient real-time LA regulation [33]. 

Fiber optic technology is a perspective alternative for traditional sensing methods. Indeed, fiber optic sensors have low heat conductivity, they are immune to electromagnetic interferences, and prone to the laser light absorption because of the fiber material (glass or polymer) [35]. The most popular fiber optic temperature measurements for TAs are divided into two types: distributed and quasi-distributed [36]. Distributed sensing relies on Rayleigh scattering phenomena and measures only the relative change of temperature profile since it analyzes the spectral shift between two states: measured and reference one (without temperature change) [37]. Currently, the main technique for thermal measurements is optical frequency-domain reflectometry (OFDR) based Luna OBR4600, which has sub-mm spatial accuracy [38]. Standard single-mode fiber is used as a cheap sensor for distributed sensing, but an expensive interrogator is needed for it. The high interrogator cost and low sampling rates make distributed sensing not well suited for real-time temperature monitoring needed for ablation regulation [35].

Quasi-distributed sensing relies on fiber Bragg grating (FBG) measurements. FBG is a structure with a periodic change of the refractive index along the fiber core. FBG acts as a wavelength-dependent reflector: incident broadband light is reflected at a specific wavelength, called the Bragg wavelength, $\lambda_{B}$ [39]. The reflected wavelength depends on the grating period (the distance between two high-index regions), which is changed during external temperature perturbations. In addition, multi-point measurements are possible when a chain of FBGs, each with a different grating period, is inscribed along the fiber core. In this case, the set of different Bragg wavelengths is analyzed to monitor the spatial temperature profile along the fiber. FBG monitoring allows lower costs of the interrogators and high sampling rates (up to 10 kHz), which makes FBG sensing more suitable for LA regulation [40].

Several studies implemented fiber optic- [36,37,41] and quasi-distributed sensing [33,42,43,44,45] to monitor temperature during LA, and showed the capability of fiber optic sensors to provide spatial maps of the tissue temperature after the treatment completion. However, none of these studies investigate the performance of fiber optic sensors for temperature-based LA regulation, where also real-time monitoring is indispensable. 

In these regards, this work focuses on the development of the first platform dedicated to real-time spatial temperature monitoring and related therapy regulation by introducing FBG-based strategy. To improve the accuracy and spatial resolution of the sensors, custom-made highly dense FBG arrays were inscribed in polyimide-coated single-mode fibers. With this sensing technique, the temperature was monitored simultaneously in 120 regions of an ex vivo liver undergoing LA. The developed closed-loop temperature control algorithm aimed at maintaining the temperature in the laser-irradiated liver area at specific set thresholds (43 and 55 °C), in correspondence to specific radii (2 and 6 mm) of the targeted zone. The obtained thermal maps and ablated tissue analysis prove the efficacy of the fabricated FBG arrays and the developed algorithm for LA controlling. Besides, thermal maps clearly show the importance of temperature threshold setting to control the spatial extension of the ablated zone and to keep the maximum temperature below critical values. 

## 2. Materials and Methods

### 2.1. FBG-Based Sensing

**Figure 1:**
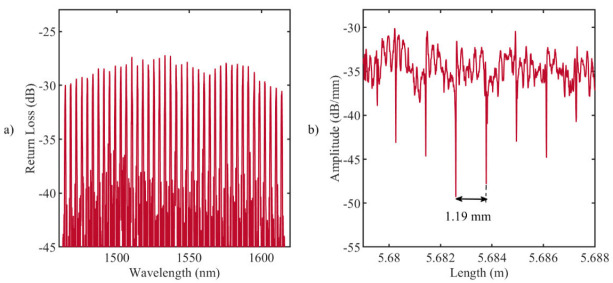
(**a**) Reflection spectrum of the fabricated fiber Bragg grating (FBG) array with the femtosecond point-by-point writing technology: 40 gratings, equidistant in 1464.5–1614.5 nm wavelength range; (**b**) LUNA OBR 4600 backscattering signal: the distance between the ends of the gratings (drops in amplitude) is 1.19 mm.

For temperature measurements, custom-made arrays of 40 FBGs were inscribed in a single-mode optical fiber SM1500(9/125)P (Fibercore Ltd., Southampton, UK) using the femtosecond point-by-point writing technology [46]. Femtosecond pulses with a wavelength of 1026 nm, duration of 232 fs, pulse repetition rate of 1 kHz, and pulse energy of ~100 nJ were produced by Pharos 6W laser system (Light Conversion Ltd., Vilnius, Lithuania) and focused into the fiber core region with a microobjective (NA = 0.65). Precise fiber positioning in the process of FBG writing was provided by ABL1000 air-bearing linear stage (Aerotech Inc., Pittsburgh, PA, USA). The design of the grating lengths has been performed to space the resulting Bragg wavelengths at each 4 nm, and to fit the spectral region (Figure 1a) of the Micron Optics si255 interrogation unit (Micron Optics, Atlanta, GA, USA), which ranges from 1460 to 1620 nm. The choice to coat the gratings with polyimide is motivated by the excellent thermal properties of this material over the standard acrylate coating, such as the high-temperature resistance up to 400 °C, and the low thermal conductivity [46,47,48]. The transparency of the polyimide coating for IR femtosecond radiation allowed us to inscribe FBG arrays through the protective coating, thus preserving temperature and mechanical performance of the fiber. The FBG arrays have grating lengths of 1.19 mm, and the edge-to-edge distances between gratings equal to 0.01 mm. The chosen length of an FBG provided a simultaneous high spatial resolution and a narrow spectral width of an individual resonance peak, which reduced the mutual influence of neighboring resonances during nonuniform heating of the array. Indeed, the maximum temperature near the laser applicator tip can overcome 300 °C and gradient can be up to 50 °C/mm [24], and the developed FBGs can measure such temperature without any interference between FBG peaks.

Figure 1b illustrates the backscattering signal measured by LUNA OBR 4600 reflectometer (9.607 µm spatial resolution): each drop in amplitude corresponds to the ends of the grating, and the distance between drops is approximately equal to 1.19 mm. The analysis of the reflected Bragg wavelength shifts, $∆\lambda_{B}$, provides information about the temperature along the grating, ∆T [49]:

(1) $$\frac{∆\lambda_{B}}{\lambda_{B}}=\frac{\lambda_{B,∆T}-\lambda_{B,initial}}{\lambda_{B}}=\alpha ∆T$$

where α (°C^−1^) is the thermal sensitivity of the grating.

The thermal sensitivity of the FBGs is (7.43 ± 0.01) × 10^−6^ °C^−1^, as obtained after static calibration in a thermostatic dry-block calibrator in the temperature range 20 to 130 °C. 

### 2.2. Experimental Setup

**Figure 2:**
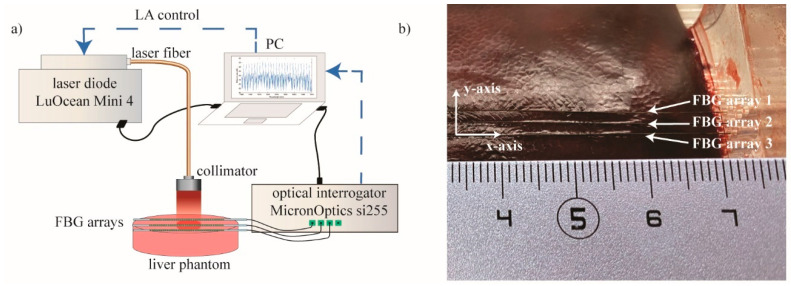
(**a**) Schematic representation of the experimental setup: superficial LA of ex vivo porcine liver. Laser control was based on FBG temperature measurements. (**b**) Picture of the plexiglass box containing the liver and allowing the 2 mm-distance arrangement of the three FBG arrays.

For ablation experiments, an 808 nm continuous wave diode laser (LuOcean Mini 4, Lumics, Berlin, Germany) emitting in the near-infrared range was used. Ablation was performed with a laser power of 5 W in a superficial manner: laser light was guided through a 440 μm diameter quartz optical fiber connected to a collimator (OZ Optics Ltd., Ottawa, ON, Canada) and positioned perpendicularly to the porcine liver surface at a 7 cm distance from it (Figure 2a). The laser beam spot diameter was 10 mm, and the ablation duration was 90 s.

Experiments were performed on ex vivo porcine liver. The liver was obtained from a local butcher the same day of the experiment and stored at 4 °C until the experiment was carried out. Three highly dense FBG arrays, (FBG arrays 1, 2, and 3) were placed on the liver surface by means of a custom-made box. The plexiglass box was used to control the relative position between each fiber, equal to 2 mm (Figure 2b). The laser spot was focused on the center of the arrays, and Micron Optics si255 interrogation unit was used to measure the reflected Bragg wavelength spectra of the array, with a sampling frequency of 100 Hz.

### 2.3. Temperature-Feedback Control Strategy for the Zone Control Logic

**Figure 3:**
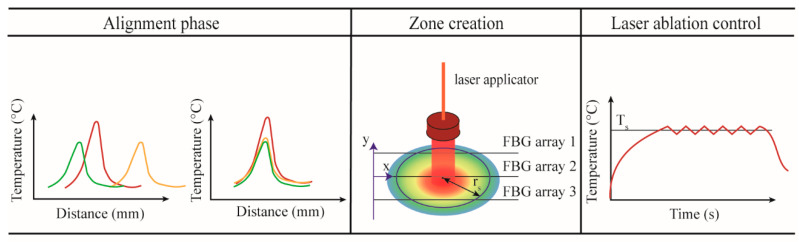
Main phases of the *zone-control logic* designed to regulate the laser ablation (LA) according to the quasi-distributed temperature measured with FBG arrays.

The developed real-time closed-loop temperature control algorithm utilizes an ON–OFF logic based on spatial temperature information, and performs a strategy that the authors call *zone-control logic*. The implemented *zone-control logic* consists of three main sub-parts: (*i*) alignment, (*ii*) creation of the spatial temperature maps and definition of the radius, and (*iii*) laser ablation control (Figure 3).

In the alignment phase, the tissue is heated up by the laser source until the maximum temperature reaches the pre-phase threshold temperature (about 33 °C). This threshold is usually reached within 4−5 s from the laser activation and has been chosen to avoid potential damage to the tissue. The profiles measured by the three arrays are aligned employing the centroid method, which finds the centers of the Gaussian distribution along *y*-axis measured by each array and shift one over the other in order to match the centers [36]. The alignment is a crucial step; indeed, when several FBG arrays are used, the alignment between the acquired temperature profiles is mandatory to correctly reconstruct the real-time temperature map.

It is important to highlight that the temperature measurements have higher spatial resolution along the *x*-axis (FBG array resolution is 1.2 mm) and lower resolution along *y*-axis (distance between FBG arrays is 2 mm). For better thermal mapping, the temperature profiles are linearly interpolated along and between the FBG arrays. Once the interpolation is performed, the thermal map is visualized in real-time during the ongoing LA procedure. From this map, the software defines the position in which the maximum temperature value is located, and, starting from this position, the user can select the radius (*r_s_*) of the circumference corresponding to the zone that has to be controlled at the set temperature *T_s_.*

In the case of uncontrolled ablation, the laser light is delivered in continuous modality. Ablation starts at room temperature *T*_0_, and the laser is ON until the moment when the laser system is switched off by the user. In the case of controlled ablation, the control logic works as follows: ablation starts at room temperature *T*_0_, and the laser is ON until the moment when the maximum temperature measured by the sensor placed at *r_s_* exceeds the set temperature *T_s_.* Then, the laser follows an ON–OFF logic to maintain a maximum temperature close to *T_s_*. The comparison of *T_s_* with the measured maximum temperature is executed each Δτ seconds. The comparing period should not be less than 1 ms to prevent pulsed-mode behavior of the laser, which may lead to other laser–tissue interaction effects, such as explosive evaporation and cavitation in the irradiated tissue [50]. The preliminary evaluation of the optimal Δτ carried out by the authors shows that Δτ = 1 s leads to smooth temperature control and a spatially confined ablation region [51]; hence, this value for the comparison period was used for all experiments presented in this work.

The experiments were performed with the settings listed in Table 1:

LA without feedback regulation (uncontrolled ablation) was considered as a reference. The *zone-control logic* was designed and implemented in LabVIEW; the program was developed to receive Bragg wavelengths data from the Micron Optics interrogation unit, reconstruct spatial temperature maps, define the values for *r_s_* and *T_s_,* and adjust the laser power based on these measurements using an ON–OFF logic in real time (Figure 3). 

## 3. Results

### 3.1. Thermal Analyses on the Temperature Profiles Measured by FBG Array 2

**Figure 4:**
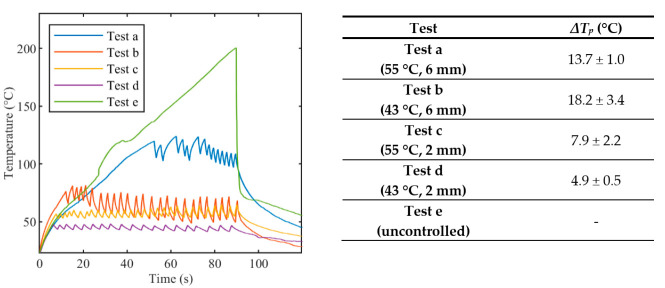
Peak temperature profiles recorded from array #2 during laser ablation: uncontrolled ablation and controlled ablation with different set temperature values *T_s_*. In the Table, oscillations *ΔT_p_* are expressed as mean value ± standard deviation.

**Figure 5:**
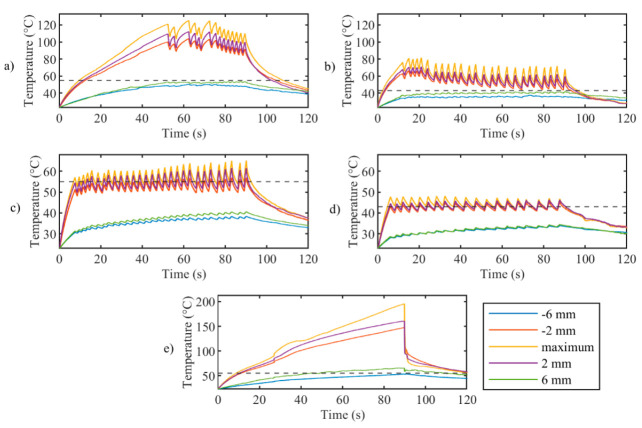
Trends in time of the maximum temperature (yellow curves) and the temperature measured at *r_s_* distances from the center (other 115 profiles are not shown for clarity of the figures) for controlled ablation carried out in (**a**) Test a, 55 °C and 6 mm; (**b**) Test b, 43 °C and 6 mm; (**c**) Test c, 55 °C and 2 mm; (**d**) Test d, 43 °C and 2 mm; (**e**) uncontrolled ablation.

**Figure 6:**
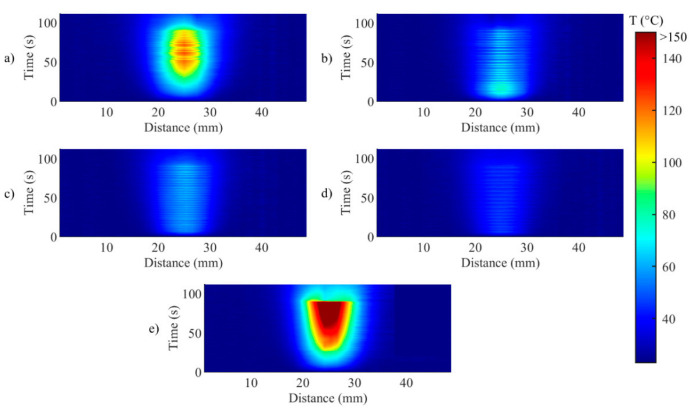
Two-dimensional thermal maps (distance along the sensor vs. time) during (**a**) Test a, 55 °C and 6 mm; (**b**) Test b, 43 °C and 6 mm; (**c**) Test c, 55 °C and 2 mm; (**d**) Test d, 43 °C and 2 mm; (**e**) uncontrolled ablation.

**Figure 7:**
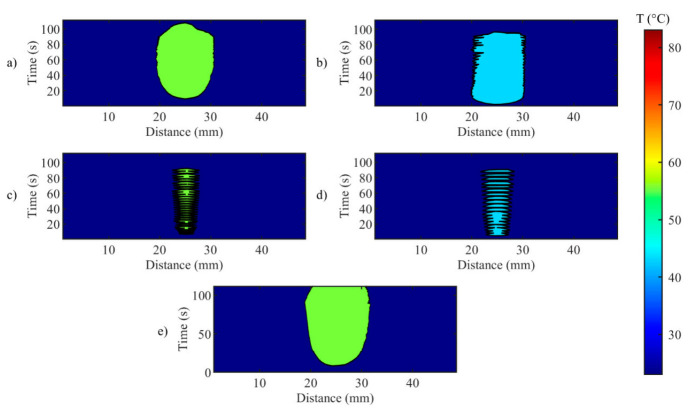
Evolution of the width of the hyperthermia zone (>43 °C) in time for during (**a**) Test a, 55 °C and 6 mm; (**b**) Test b, 43 °C and 6 mm; (**c**) Test c, 55 °C and 2 mm; (**d**) Test d, 43 °C and 2 mm; (**e**) uncontrolled ablation.

The first analyses on the effect of the *zone-control logic* on the LA outcome are performed considering the temperature profiles measured by the 40 gratings of the FBG array 2 experiencing the highest temperature along the *y*-axis. The results of these analyses are illustrated in Figure 4, Figure 5, Figure 6 and Figure 7.

Figure 4 reports tissue temperatures during LA measured in correspondence to the grating of FBG array 2 experiencing the highest temperature. The profiles obtained in the controlled cases (Figure 4, blue, red, yellow, and purple lines) are distinguishable from the uncontrolled experiment. (Figure 4, green line). In particular, the experiments performed with the temperature-feedback control show that the maximum temperatures follow the set thresholds; moreover, a characteristic sawtooth-like shape is observed, due to the effect of the laser that is switched ON and OFF according to the set *T_s_* and *r_s_*. In each experiment, these maximum temperatures oscillate around the correspondent *T_s_*, and the magnitude of these oscillations depends on *r_s_*. The table enclosed in Figure 4 lists the values of these oscillations, *ΔT_p_*, which are calculated by the difference between the temperature at the highest peak and the subsequent valley. 

In the case of uncontrolled ablation, tissue temperature achieves 200 °C after 90 s of ablation, showing an irregular trend during the time and no oscillations.

Figure 5 presents the maximum temperature profile (yellow lines) and the profiles measured by the sensor placed at the set *r_s_* on FBG array 2. It clearly illustrates that oscillations decrease with distance from the maximum temperature for controlled ablation cases; this happens because, at a certain distance from the laser spot, the temperature elevation due to heat conduction is predominant over the intermittent effect of the control logic. Figure 5 also confirms the proper placement of the FBG arrays with respect to the laser beam, showing the symmetry of the temperature profiles measured at *r_s_* of +/−6 mm (blue and green curves) and *r_s_* of +/−2 mm (red and purple curves). 

Figure 6 illustrates the temporal evolution of the temperature profile measured by the FBG array 2, for both controlled (Figure 6a−d) and uncontrolled ablations (Figure 6e). This representation highlights the effect of the control strategy on both the maximum temperature and on the unidimensional distribution of the temperature, in correspondence to the central axis of the ablated region. For uncontrolled ablation, after the start, the temperature increase causes the heat distributing towards edges of the ablated area with less regular behavior than controlled cases; when ablation is over, the temperature starts to decrease, but the heat dissipation is continuing towards the edges. 

Figure 6 anticipates that the edges of the ablated zones are not uniform, as further analyzed in Figure 7, which depicts the evolution of the width of the hyperthermia zone (>43 °C) in time. It is worth mentioning that the zone is small and not continuous for the cases *T_s_* = 55 °C, *r_s_* = 2 mm and for the case *T_s_* = 43 °C, *r_s_* = 2 mm (Figure 7c,d). The discontinuities shown in the mentioned cases are due to temperature oscillations occurring around *T_s_*. This phenomenon happens because *r_s_* is chosen close to the center of the circumference, and when *T_s_* was reached, the laser was set immediately OFF, not allowing broadening of the hyperthermia zone. These results highlight the relevance of the size of the zone to be controlled, and the effect of *r_s_* on the temperature distribution experienced by the biological tissue.

### 3.2. Thermal Analyses on the Spatial Temperature Distribution

**Figure 8:**
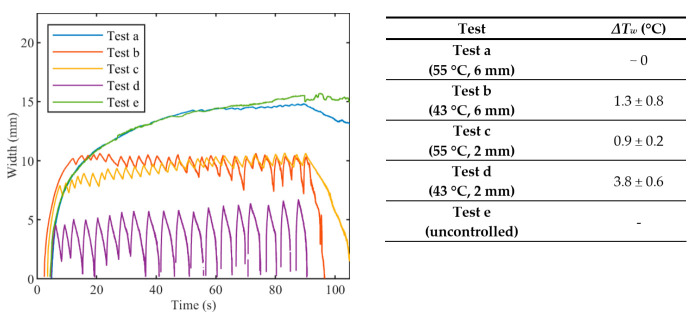
Width of hyperthermia zone (>43 °C) vs. time for controlled ablations with different set temperatures *T_s_* and radii *r_s_*, and for uncontrolled ablation. In the table, oscillations are expressed as mean value ± standard deviation.

**Figure 9:**
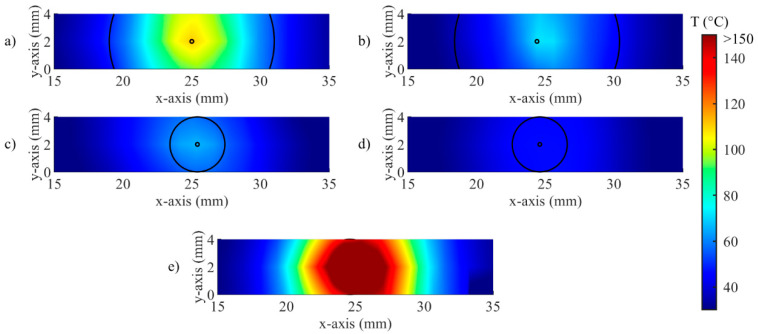
Visualization of the real-time temperature maps and zone control circumferences at t = 90 s for (**a**) Test a, 55 °C and 6 mm; (**b**) Test b, 43 °C and 6 mm; (**c**) Test c, 55 °C and 2 mm; (**d**) Test d, 43 °C and 2 mm; (**e**) uncontrolled ablation.

**Figure 10:**
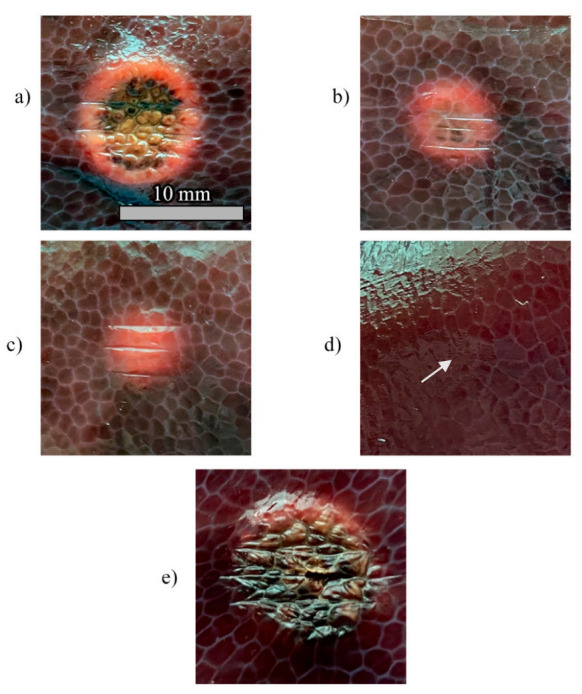
Pictures of the tissue damage produced by the different control strategies for (**a**) Test a, 55 °C and 6 mm; (**b**) Test b, 43 °C and 6 mm; (**c**) Test c, 55 °C and 2 mm; (**d**) Test d, 43 °C and 2 mm; (**e**) uncontrolled ablation.

**Figure 11:**
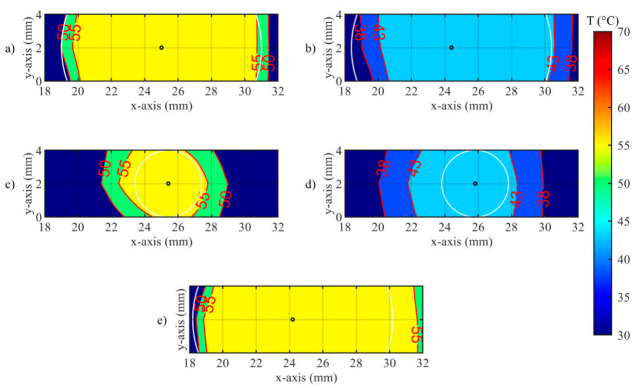
Isotherms of spatial distribution measured on the tissue when laser changes its state from ON to OFF, for (**a**) Test a, 55 °C and 6 mm; (**b**) Test b, 43 °C and 6 mm; (**c**) Test c, 55 °C and 2 mm; (**d**) Test d, 43 °C and 2 mm; (**e**) uncontrolled ablation.

**Figure 12:**
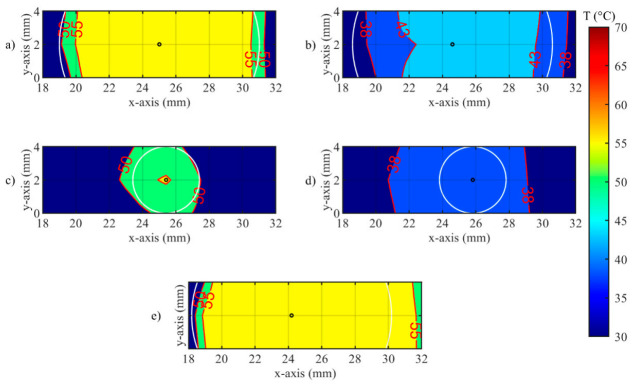
Isotherms of spatial distribution measured on the tissue when laser changes its state from OFF to ON, for (**a**) Test a, 55 °C and 6 mm; (**b**) Test b, 43 °C and 6 mm; (**c**) Test c, 55 °C and 2 mm; (**d**) Test d, 43 °C and 2 mm; (**e**) uncontrolled ablation.

Detailed analyses on the effect of the *zone-control logic* on the LA outcome are performed considering the spatial temperature distributions measured by all the 120 gratings. The results of these analyses are illustrated in Figure 8, Figure 9, Figure 10, Figure 11 and Figure 12.

The widths of the contours at 43 °C are presented in Figure 8. Both *T_s_* and *r_s_* affect the width of the hyperthermia zone and the fluctuations of the width. Indeed, the width at 43 °C observed in Test a (*T_s_* = 55 °C, *r_s_* = 6 mm) is close to the width at 43 °C observed in Test e (uncontrolled ablation), and both reach a value of about 15 mm at the end of the thermal procedure. Due to the effect of the heat conduction, after that the laser is switched OFF, the Test a and uncontrolled case show different behavior: the width at 43 °C for the uncontrolled case remains almost constant whereat it starts to drop off immediately for Test a. The widths at 43 °C achieved with Test c (*T_s_* = 55 °C, *r_s_* = 2 mm) and Test b (*T_s_* = 43 °C, *r_s_* = 6 mm) presents similar trends, with a maximum value of about 10 mm at the end of the ablation. For the Test d (*T_s_* = 43 °C, *r_s_* = 2 mm), fluctuation of the width at 43 °C reaches approximately 7 mm, corresponding also to the maximum value at the end of the procedure. The Table enclosed in Figure 8 lists the values of the oscillations of the width (*ΔT_w_*), which are calculated by the difference between the temperature at the highest peak and the temperature at the subsequent lowest peak.

An example of the real-time visualization provided by the developed software is given in Figure 9. Here, the spatial distribution of temperature measured by the FBGs is updated during the procedure, and the circumference defined according to *r_s_* are overlapped. The entire circumference is represented for *r_s_* = 2 mm (Figure 9c,d), because the distance between arrays is 2 mm; in the case of *r_s_* = 6 mm (Figure 9a,b), only two arcs of the circumference can be traced. As expected, no circumference is illustrated for the uncontrolled case (Figure 9e). 

Thermal damages obtained in the hepatic tissue with controlled and uncontrolled ablations are presented in Figure 10. The RGB images show that the damage is visible for *T_s_* = 55 °C, regardless of the value of *r_s_* (Figure 10a,c), and that the damaged area increases with *r_s_*. In the experiments carried out at *T_s_* = 43 °C, the thermal damage is distinguishable only for *r_s_* = 6 mm (Figure 10b), whereas a minor effect of tissue dehydration is slightly noticeable for *r_s_* = 2 mm (Figure 10d, white arrow). As expected, the high temperature (up to 200 °C) experienced by the tissue during uncontrolled ablation (Figure 10e) caused the largest damaged area as well as the more severe damage. It is worth highlighting that the uncontrolled ablation margins of the lesion are more irregular than the margins obtained with the *zone-control logic*. 

A closer look at the effect of the developed *zone-control logic* is provided by Figure 11 and Figure 12. In these illustrations, the effect of the oscillations previously presented for single fiber measurements is observable on the spatial temperature distribution. Figure 11 and Figure 12 show two specific moments of the implemented temperature feedback regulation, i.e., when the control logic sets the laser from ON to OFF (Figure 11) and from OFF to ON (Figure 12). In the figures, the isothermal regions between the set *T_s_* and *T_s_*-5 °C are shown for each test.

Figure 11 illustrates the isotherms in correspondence of the peak of overshooting (here, laser changes its state from ON to OFF). Indeed, the isotherms of set temperatures reach the set radii, and, in some points, exceeds it. The value of the radius affects the overshooting area: indeed, for *r_s_* = 2 mm, the isotherms at both set *T_s_* are larger than the set circumference.

In Figure 12 the undershooting situation is presented (laser state changes from OFF to ON). Here, the isotherms of set temperature are distant from the set radius, moreover for test d, no region at 43 °C is detected.

The time evolutions of the spatial temperature maps (thermal and isothermal) for controlled and uncontrolled cases (Test b and e, correspondingly) are reported in the Supplementary Video. The video illustrates the effect of the set parameters on the heat distribution during the ablation procedure.

## 4. Discussion

This work originally presents a real-time closed-loop temperature control strategy, called *zone-control logic*, for controlling and tuning the laser ablation outcome in biological tissues. The temperature feedback control strategy is based on a custom-made software providing real-time monitoring of the spatial temperature distribution measured by a network of 120 FBGs. FBG arrays with high spatial resolution properties (1.2 mm distance between centers of consecutive gratings) and high-temperature resistance coating were fabricated with the femtosecond point-by-point writing technology [46]. LabVIEW software was used to design and implement the ON–OFF program that regulates the mode of operation of the laser source with the temperature measured in the hepatic tissue. The user interface of the software allows clinician to set two parameters of the procedure, i.e., the radius of the zone to be controlled and the specific temperature, that will be controlled automatically during the procedure. The choice of the parameter values should be based on individual patient’s needs, i.e., the size of the tumor obtained from pre-operative images, or typical temperature settings known from the literature. In addition, the real-time temperature mapping is also provided on the user interface.

The results of this work show that the implemented strategy is suitable to achieve the desired control of the ablated area, by opportunely tuning the radius and the set temperature. The set temperature was contained within the proper range, corresponding to specific thermal states of biological tissues inside the low-temperature damage accumulation process [52,53]. Only a few previous studies presented the real-time control of the laser settings according to the tissue temperature, and most of them employed single-point measurements [28,31,51], or contactless thermometric systems [25]. Conversely, our *zone-control logic* allows for a multipoint control of the tissue temperature, which can be adapted according to the size of the desired tissue region to be treated. With the proposed unique approach, the choice of the radius and the temperature threshold can be adapted to the specific needs of the therapy. Our results demonstrated that the width at 43 °C close to 15 mm can be reached by keeping the ablation zone with radius 6 mm at 55 °C for 90 s, obtaining a spatial thermal effect similar to the one achieved with uncontrolled ablation, but in safer conditions. Indeed, when continuous laser irradiation is used, tissue temperature rapidly increased to high values (200 °C) due to the constant supply of laser energy, with potential risks for the organ. 

The Figure 6, Figure 7 and Figure 8 confirm the effect of the control logic on the extension of the thermal margins of the zones, and the effectiveness of having real-time monitoring to observe the evolution of the treatment (Figure 9). Figure 10 further proves the relevance of the *zone-control logic* to contain the damage to the interested area and to achieve regular margins, clearly showing the different thermal outcome in RGB images obtained after uncontrolled and controlled ablations.

The multi-point temperature measurement is enabled thanks to highly dense FBG arrays. The main advantages over other temperature monitoring methods for LA control (thermocouples, thermistors, and photo-optic probes) [28,29,30] are the following: quasi-distributed sensing capability, minimal invasiveness, and no self-heating, that make them well suited for LA applications. The quasi-distributed monitoring property of FBGs allows for high-spatial-resolution temperature measurements along the fiber and accurate evaluation of the temperature distribution. These peculiarities make the FBGs the good candidates to scale the implemented strategy to control also interstitial laser applications [44].

It has been observed, and further confirmed by this work, that the ON–OFF strategy leads to oscillations of the maximum temperature values across the set threshold [51]. The oscillations contribute to the thermal history of the treatment, due to the heat that remains in the tissue after the laser is turned off and to the cooling occurring between consecutive irradiations [25]. Figure 5 shows that these oscillations are marked in correspondence of the center of the ablation zone, but their amplitudes significantly decrease with the radius of the zone under control, providing smoothly increasing temperature trends at 6 mm from the center of the ablation zone (Figure 5, blue and green curves). Indeed, since the irradiation is repeated before complete cooling of the medium, the elevations of the tissue temperature can be additive [25], but slower than the temperature increase produced by continuous laser (uncontrolled ablation). A further improvement of the control strategy towards the extinguishment of the oscillations can be based on proportional–integral–derivative (PID) control, which has been proved to be effective mostly for single-point measurement [54] and in adaptive control systems [55]. 

## 5. Conclusions

This work presents a novel *zone-control logic*, aimed at controlling the outcome of LA in biological tissues through the temperature measured by highly dense FBGs arrays. The implemented strategy and monitoring system provide laser thermal treatment with the ability to maintain a controlled temperature in the targeted area. The results of this study encourage further investigation of the optimal control-loop and laser system settings for improving LA effects. This study provides the foundation for the use of a control strategy for optimizing laser ablation and obtaining predictable outcomes in a clinical scenario, which still requires the support of the technology to provide a reliable endpoint for the treatment efficacy.

## Figures and Tables

**Table 1:** Settings used for the experiments: for the temperature-based controlled ablations (*zone-control logic*) set temperature $T_{S}$ and radius (*r_s_*) of the circumference of the zone under control are defined.

Setting	Test a	Test b	Test c	Test d	Test e
$T_{S}\;(\text{°}C)$	55	43	55	43	uncontrolled
*r_S_* (mm)	6	6	2	2	

## Supplementary Materials

The following are available online at https://www.mdpi.com/1424-8220/20/22/6496/s1, Video S1: Spatial thermal map (x- and y-axis) evolution during the laser ablation experiments. In the video, the controlled ablation experiment (Test b) is on the top; the uncontrolled experiment (Test e) is on the bottom. Two types of maps are provided: isothermal and thermal distributions. The 6 mm radii are marked with black circles for the controlled and uncontrolled cases.
